# Prophylactic Antibiotics for Prevention of Infective Endocarditis or Bacteremia in Pediatric Patients With Congenital Heart Disease Undergoing Dental Procedures: Systematic Review

**DOI:** 10.1111/scd.70112

**Published:** 2025-11-21

**Authors:** Cameron Loper, Jordan Baskette, Timothy R. Fagan, Divesh Sardana

**Affiliations:** ^1^ DDS Candidate, College of Dentistry Oklahoma University Health Sciences Center (OUHSC) Oklahoma City Oklahoma USA; ^2^ William E. Brown Chair in Dentistry Department of Developmental Sciences College of Dentistry Oklahoma University Health Sciences Center (OUHSC) Oklahoma City Oklahoma USA; ^3^ Assistant Professor, Department of Pediatric Dentistry Indiana University School of Dentistry, and James Whitcomb Riley Hospital for Children Indianapolis Indiana USA

**Keywords:** antibacterial agents, antibiotic prophylaxis, antimicrobial stewardship, bacteremia, endocarditis, heart

## Abstract

**Aim:**

To assess the effectiveness of prophylactic antibiotics in preventing infective endocarditis (IE) after dental procedures in pediatric patients with congenital heart disease.

**Methods:**

Six electronic databases and grey literature were searched for randomized controlled trials (RCTs), case‐control studies, and cohort studies in pediatric patients who received antibiotic prophylaxis (AP) before any dental procedure compared to pediatric patients undergoing the same procedures without AP to prevent IE and/or bacteremia. The Newcastle–Ottawa scale was used to evaluate the internal validity of the included studies. The Grading of Recommendations Assessment, Development, and Evaluation (GRADE) was used to assess the certainty of evidence.

**Results:**

1774 studies were screened after duplicate removal. Three case‐control studies met the inclusion criteria and were included. One study evaluated IE as an outcome, whereas the other two studies evaluated bacteremia after dental procedures. Meta‐analysis could not be conducted for IE, whereas pooling results were attempted for two studies evaluating bacteremia as an outcome. The odds of IE after a dental procedure within the last 6 months with AP were higher than without AP (Odds ratio: 3.44, 95% CI: 0.93, 12.65, *p* = 0.06) in the only study. However, AP was effectively able to reduce bacteremia after dental procedures in two studies that evaluated bacteremia (Pooled odds ratio: 0.24, 95% CI: 0.14, 0.42, *p* < 0.05).

**Conclusions:**

The GRADE evidence was very low to conclude that prophylactic antibiotics might have a role in preventing IE or bacteremia in children with congenital cardiac diseases due to insufficient studies.

## 1 Introduction

The oral cavity harbors numerous microorganisms, some of which are opportunistic and have the potential to cause systemic and local infections when the host's immunity is compromised or the growth conditions for those microbial species are favorable. It has been well known that activities of daily living (toothbrushing and flossing) and invasive dental procedures (ranging from scaling to extraction) are known to cause transient bacteremia [1, 2], a term that implies the transient presence of viable bacteria in the circulating blood. The bacteria normally clears from the circulating blood in individuals with healthy immune response and cannot adhere to healthy tissue surfaces; however, in individuals with structural defects (like rheumatic heart disease) or unhealthy tissue surfaces favorable for adherence (prosthetic joint replacement, heart valve replacement, congenital heart disease), there has been ongoing concerns and discussions that these bacteria have a high likelihood of adhering and colonizing on these favorable surfaces and thus causing systemic infections. This adherence often leads to more serious complications like infective endocarditis (IE) or periprosthetic joint infections [3, 4]. Thus, antibiotic prophylaxis (AP) before dental procedures has been recommended by the American Heart Association (AHA) since 1955, and has undergone many revisions since then, with the notable amendments in 1997, 2007, and 2021. With advancements in our understanding of the role of AP in preventing systemic infections, there have been numerous changes over the past few decades, including modifications in drug regimens and dosages, as well as revisions to clinical indications pertaining to the dental procedures and the underlying systemic disorders. Systemic conditions that once warranted AP under earlier guidelines have since been removed in updated recommendations by the AHA and the American Dental Association (ADA) [5, 6, 7]. This paradigm shift has been due to a better understanding of the etiopathogenesis of IE as well as findings from recent studies that have underscored the adverse effects of antibiotics, the risk of antibiotic resistance, and the fact that cost‐effectiveness often outweighs the beneficial effects of AP. Although the guidelines on AP before dental procedures to prevent IE have evolved, AP is still indicated and recommended for many dental procedures and cardiac conditions. The National Institute for Health and Care Excellence (NICE) UK guidelines (2008) reignited the debate, as these guidelines no longer recommended AP before dental procedures in high‐risk cardiac patients. However, to add to the confusion, the NICE guidelines were modified in 2015, where the word *“routinely”* was added to indicate *“Antibiotic prophylaxis against infective endocarditis is not recommended* ***routinely*** *for people undergoing dental procedures”* [8]. The 2015 change was due to concerns that the cases of IE had been rising in England since the 2008 guidelines came into effect, and in turn, the prescription of antibiotics decreased [9]. The retrospective study estimated that there were nearly 35 more cases of IE after the NICE 2008 guidelines (**cessation of AP** before dental procedures in high‐risk patients) than if the 2008 guidelines had not been adopted and the historical trend (**AP before dental procedures** in high‐risk patients) had continued. Concerns may have been raised because of the confounding variables, study design, and rise in IE cases after dental procedures without AP in patients with no or low cardiac risk, but the authors cautioned temporal association between a change in antibiotic prescription and the incidence of IE.

With the ongoing debate, confusion, and change in guidelines about AP before dental procedures in high‐risk cardiac patients, it has been shown that despite the change in guidelines of that country, healthcare providers and dentists might continue to give AP even when prophylaxis is not indicated or necessary in the most recent guideline [3, 10, 11]. Although there have been systematic reviews that have assessed the evidence on the use of AP before dental procedures in high‐risk cardiac patients, all of them have focused on IE as an outcome in adults or included children as a subgroup in their analysis for a wider and paramount scientific topic. Thus, the purpose of the present systematic review was to assess and synthesize the evidence regarding the effectiveness of AP before dental procedures in pediatric patients with congenital cardiac disorders for the prevention of IE. Also, the secondary aim was to synthesize the evidence regarding the use of AP in preventing bacteremia after dental procedures in pediatric patients with congenital cardiac disorders compared to controls not receiving AP.

## 2 Materials and Methods

### 2.1 Protocol and Registration

The methodology of the current review was formulated in advance by adhering to the Cochrane Handbook and documented in the protocol [12]. Subsequently, the protocol was designed a priori and registered at the International Prospective Register of Systematic Reviews (PROSPERO ID: CRD42024605783). The review is being reported as per the PRISMA (Preferred Reporting Items for Systematic Reviews and Meta‐Analyses) statement and checklist [13].

### 2.2 Study Eligibility Criteria

The articles in the present review included randomized controlled trials (RCTs), case‐control studies, and cohort studies that compared pediatric patients with congenital heart diseases (CHD) who had undergone dental treatment with AP versus the patient population who did not receive AP. The detailed PECO (Population, Exposure, Control, and Outcome) schema for inclusion and exclusion criteria is outlined in Table 1.

**TABLE 1:** PECOS/PICOS schema for the eligibility criteria.

Study design (*S*)	Randomized clinical trials (PICOS)	Case‐control studies (PECOS)	Cohort studies (PECOS)
Population (*P*)	Pediatric patients with CHD (low, moderate, or high risk of IE)	Pediatric patients with CHD (low, moderate, or high risk of IE)	Pediatric patients with CHD (low, moderate, or high risk of IE)
Intervention (*I*)	AP before any dental procedure	—	—
Exposure (*E*)	—	AP before the dental procedure	AP before the dental procedure
Control (*C*)	Patients who did not receive AP before any dental procedure	Patients who did not receive AP before any dental procedure (normal or cardiac patients)	Patients who did not receive IE prophylaxis before any dental procedure (normal or cardiac patients)
Outcome (*O*)	Incidence of IE (primary outcome) or bacteremia (secondary outcome)	Incidence of IE (primary outcome) or bacteremia (secondary outcome)	Incidence of IE (primary outcome) or bacteremia (secondary outcome)

### 2.3 Information Sources and Literature Search

Six electronic databases were systematically searched by two independent authors (S.D. and L.C.) with no start date restrictions or language restrictions, up to and including April 2024, using the broad MeSH terms and keywords. The databases searched were as follows: Web of Science, Medline (via Ovid), Embase Classic, Scopus, CINAHL, and PubMed [Appendix 1]. Additionally, cross‐references of the included articles were hand‐searched for any potentially relevant articles meeting the eligibility criteria. An online search on Google was attempted with broad keywords to identify any unpublished theses or conference abstracts, or any other records not captured by electronic databases. The search was updated manually in May 2024 by hand‐searching.

### 2.4 Study Selection

The results obtained through the search of the databases were systematically managed using EndNote 20.6 software for Windows (Clarivate Analytics, Philadelphia, USA) and then exported to Covidence (Covidence SaaS Platform, Melbourne, Australia). After removing duplicates automatically, two authors (S.D. and L.C.) independently screened the titles and their respective abstracts in a standardized manner to decide upon their inclusion for full‐text reading as defined by the preset inclusion criteria. Full‐text reading was performed by two authors (S.D. and B.J.) for the articles that did not provide clear information about the study methodology or were considered potential inclusions based on the eligibility criteria (Table 1). Cohen's kappa coefficient (*κ*) was computed to determine the level of inter‐rater agreement among two reviewers after full‐text reading. Incongruities over the final inclusion were discussed among the authors, and if required, the third author (L.C.) acted as an arbiter.

### 2.5 Data Items

Information and data pertaining to the following parameters were extracted from each study: author, year of study, country of study, mean age and/or range, number of participants, the type of study, antimicrobial agents used (if applicable), and clinical procedures performed. Outcome data considered potentially relevant for the review included incidences of bacteremia and IE in the AP and non‐AP groups following any dental procedure. Data on incidences of bacteremia and IE following specific dental treatments (extractions, cleaning, home care) with and without AP were also found to be relevant.

### 2.6 Risk of Bias in Individual Studies

The methodological quality of the included studies was assessed using the Newcastle–Ottawa scale (NOS) for case‐control studies (maximum of 10 stars) by two authors independently (B.J. and S.D.). Any disagreements regarding the assessments were mutually discussed to achieve consensus, and an opinion from a third reviewer (L.C.) was sought if necessary. The tool assessed the risk of bias in studies across the following domains: selection (maximum four stars), comparability (maximum two stars), and outcome (maximum four stars), and the stars were assigned if the criteria for the particular domain were met. The study was awarded one * (star) each if the definition of the cases was adequate (at‐risk pediatric cardiac patients who received AP as per our inclusion criteria), cases were representative of the cases from the population, definitions of controls were adequate, and controls were representative of the general pool of the healthy population. If the study strictly controlled for the most important factor (defined in our review as *“Antibiotic prophylaxis*),” it was awarded one star, and if it controlled for other confounding factors that could impact the AP effectiveness (for example, nonroutine antibiotics or dosing), it was awarded an additional star in the domain of comparability. The study was awarded a star each if there was a robust ascertainment of the exposure in cases and controls using the same criteria, and additionally, the study accounted for the nonresponse rates. Detailed criteria for the NOS can be accessed elsewhere [14].

### 2.7 Summary Measures and Methods of Analysis

The outcomes of this review were binary; presence or absence of IE was considered as a primary outcome, whereas presence or absence of bacteremia was considered as a secondary outcome, both reported with their corresponding 95% confidence intervals (CI). A fixed effects Mantel–Haenszel model was planned if the number of included studies was less than or equal to three, and random effects REML (restricted maximum likelihood) was planned for more than three studies, with a sensitivity analysis using a leave‐one‐out approach. Adequately homogeneous and similar outcomes in the included studies were planned to be analyzed quantitatively by pooling the results appropriately. Two authors (L.C. and B.J.) independently extracted the data on a pilot proforma, and any discrepancies were sorted out by mutual discussion or consultation with the third author if required. Statistical heterogeneity of the included studies was planned to be evaluated using the *χ* ^2^‐based *Q*‐statistic method and *I* ^2^ measurement, with significance indicated by *p* < 0.05. All the statistical analyses were done using Stata for Windows (Stata 18.0 SE Edn, College Station, TX, USA). All data generated or analyzed during this study are included in this published article and its supplementary materials 1 and 2.

## 3 Results

### 3.1 Study Selection

**FIGURE 1:**
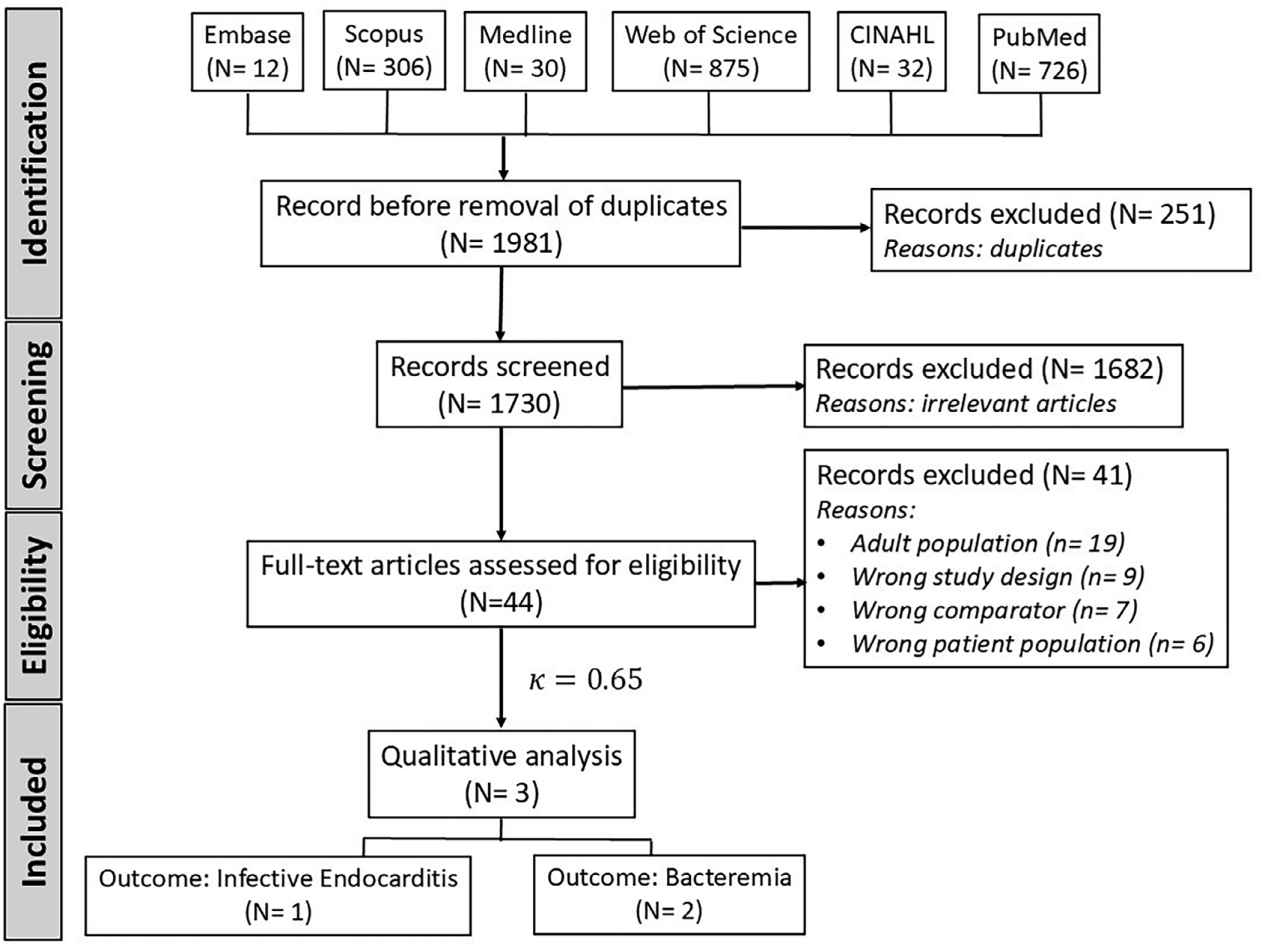
PRISMA flow chart of the study selection process.

Figure 1 provides a PRISMA flow summary of the various phases in the study selection process. After electronic database searching, a total of 1981 records were identified, but 1730 were screened for titles and abstracts after duplicate removal. Forty‐four studies were determined to be potentially eligible for the present review and were included in the full‐text reading, but finally, only three were included [15, 16, 17]. The value of *κ* at the full‐text reading stage was determined to be 0.65, indicating a moderate level of agreement.

### 3.2 Study Characteristics

Two [16, 17] of the three studies were conducted in Ireland and evaluated the risk of bacteremia after dental extractions in pediatric patients with congenital cardiac disorders. One study [15] was conducted in Taiwan and represented a large sample of 24,729 children with congenital heart disease who were followed longitudinally until the occurrence of IE or death (nested case‐control design). The dental procedures described in this study were described as extractions, endodontic procedures, periodontic procedures, and other surgical interventions involving the oral cavity. A descriptive summary of the included studies is given in Table 2.

**TABLE 2:** Characteristics of the included studies.

S. no.	Author (Year) Country of primary author	Sample size (*N*)	Mean age/Age range	Dental procedure	Antibiotic used	Dosage and administration of antibiotics	Methods of assessing outcome	Results	Funding
1.	Elliott and Dunbar [16] Ireland	217 patients (100 with no cardiac abnormalities and receiving no antibiotics 100 with cardiac abnormalities and needing AP 17 children with no cardiac abnormalities but receiving AP)	Mean age: Group 1 6.6 years Group 2: 7.5 years Group 3: 5.4 years Range: 2–13 years	Dental extractions	Penicillin was primarily used unless that was a contraindication (when erythromycin, ampicillin, or tetracycline was used).	51 patients received benzylpenicillin ½ mega approx. ½–1 h before the procedure. 22 patients received penicillin V orally, 125–250 mg and 6 h, three doses pre‐operative. Six patients received procaine penicillin at 3 00 000 units every 4–5 h pre‐operatively. Five patients received procaine penicillin and penicillin V orally at 250 mg every 6 h in two doses pre‐operatively. One patient received procaine penicillin and benzylpenicillin ½ mega given 3 days pre‐operatively. Three patients received tetracycline hydrochloride 250 mg every 6 h for 3 days pre‐operatively. Five patients received erythromycin at 125–250 mg every 6 h for three doses pre‐operatively. Two patients received ampicillin. Five patients received other antibiotic dosages and administration.	Bacteremia. Blood was collected 2–5 min after tooth manipulation, cultured in a brain heart infusion medium, and incubated for 6 days. Subcultures were tested for *α*‐hemolytic streptococci and antibiotic sensitivity.	In Group 1, 36% (*n* = 36) of children had positive cultures for *α*‐hemolytic streptococci. In Group 2, 11% (*n* = 11) had positive cultures. In Group 3, 35% (*n* = 6) yielded *α*‐hemolytic streptococci.	Not mentioned
2.	Coulter et al. [17] Ireland	58 patients (26 patients with known cardiac abnormalities and at risk for IE needing AP, 32 non‐cardiac patients not needing AP)	Mean age: 7 years Range: 2–13 years	Dental extractions	IM penicillin (eight patients) PO amoxicillin (eight patients) IV amoxicillin (six patients) IV erythromycin (four patients)	Based on recommendations of *“Endocarditis Working Party of the British Society for Antimicrobial Chemotherapy (1982 and 1986).”*	Bacteremia. A 5‐mL sample was drawn 1–2 min post‐extraction, with 1 mL placed in BACTEC broth and 2 mL in a vial containing anticoagulant. Blood was cultured using pour plates with fastidious anaerobic agar and incubated anaerobically or aerobically. Sensitivity to antibiotics was tested using microdilution broth for penicillin, amoxicillin, and erythromycin.	Of the 32 non‐cardiac patients, 20 of them had positive cultures, whereas 9 of the 26 cardiac patients were positive (*p* < 0.05).	Research Grant (Eastern Health and Social Services Board of Northern Ireland)
3.	Sun et al. [15] Taiwan	237 patients with newly diagnosed IE were identified, and a control group of 4,725 patients was randomly selected from a larger sample of children with CHD who did not have IE.	While the exact mean age is not stated, the age range of the children included suggests that the cohort likely consisted of a broad range of young children, with a particular focus on those under 3 years old at diagnosis.	Extraction, periodontics, endodontics, oral surgery, and other operations involving the oral cavity.	Antibiotic type and dosage were not specified.	Dosage not given.	Development of IE. The study used retrospective data analysis with a matched nested case‐control design to assess the association between various CHD lesions, invasive procedures, and dental procedures (with or without AP) and the incidence of IE in children with CHD.	Among the cardiac patients who developed IE (*n* = 237), 4.22% (*n* = 10) had AP before dental procedures, whereas 1.27% (*n* = 3) had dental procedures without AP. In contrast, 3.32% (*n* = 157) of the cardiac patients received dental procedures without prophylaxis and did not have IE, whereas 3.05% (*n* = 144) received AP and did not develop IE.	This study was supported by grants CTH 106A‐2A12 (Cardinal). Tien Hospital, Taiwan) and National Health Research Institutes (PH‐105‐SP‐08).

Abbreviations: CHD, congenital heart disease; IE, infective endocarditis.

### 3.3 Risk of Bias within Studies

The internal validity and the risk of bias of the three studies using the NOS are given in Table 3. None of the studies used an adequate and acceptable definition of IE or bacteremia and thus were not given enough stars in the domain of case definition. Also, the controls were hospital controls and thus no star was awarded in the domain of selection of controls. The two studies [16, 17] that evaluated bacteremia as an outcome studied patients with no known cardiac lesions as controls (probably due to ethical reasons) and were not awarded stars in the comparability domain.

**TABLE 3:** Quality assessment of the included studies using the Newcastle–Ottawa scale [14].

Author (Year)	Selection	Comparability	Exposure	Total
Elliott and Dunbar [16]	**	*	***	6/9
Coulter et al. [17]	*	*	***	5/9
Sun et al. [15]	***	**	***	8/9

### 3.4 Results of Individual Studies

The study [15] that evaluated IE as an outcome concluded that dental procedures do not increase the risk of IE irrespective of the use of AP before the dental procedure. The two studies that evaluated bacteremia as an outcome [16, 17] found AP before dental procedures in pediatric patients with congenital cardiac disorders to be significantly effective in reducing the incidence of bacteremia compared to healthy individuals not receiving AP before dental procedures.

Coulter et al. [17] sampled 26 randomly selected children at risk for IE, requiring AP, from an outpatient extraction clinic. These children were ages 2–13 years. Thirty‐two patients not at risk for IE (with no congenital cardiac abnormalities), and not requiring AP, also aged 2–13, were then randomly selected as the control group. An approximately equal number of total tooth extractions were performed in each group (a higher number of teeth per patient were extracted from the AP group). IM penicillin, oral amoxicillin, IV amoxicillin, or IV erythromycin were given to the AP group. A blood sample was taken and cultured from all patients after extractions were performed (no pre‐extraction cultures were taken). Sixty‐three percent of non‐AP patient cultures were positive for IE‐causing bacteria, while 35% of cultures from the AP group contained IE‐causing bacteria; however, 80% of all cultured colonies produced 1–2 colonies. No relationship was demonstrated between the number or type of teeth extracted and incidences of bacteremia; all patients had scrapable plaque and slight gingival inflammation, so no relationship could be drawn between plaque index and bacteremia incidences. Overall, this study found AP to be an effective measure in reducing bacteremia in patients at risk for IE.

Elliot and Dunbar [16] studied three groups of children; the first of which contained 100 healthy children not requiring AP, the second group contained 100 children with known cardiac conditions requiring AP, and the third group contained 17 children who required AP for active oral infections. Data from groups one and two were extracted as cases and controls, respectively, for the present systematic review, as these two groups met the eligibility criteria. All AP patients were treated with IM benzylpenicillin unless contraindicated, in which case they were given erythromycin, ampicillin, or tetracycline. Blood samples were taken 2–5 mins after gingival manipulation had occurred. This study only tested for alpha‐hemolytic streptococci, finding that group one had 36% positive cultures, group two had 11% positive cultures, and group three had 35% positive cultures. After statistical analysis, this study found AP to be an effective measure in reducing bacteremia in at‐risk cardiac patients, therefore lowering their risk of contracting IE.

Sun et al. [15] only included children with CHD; this was achieved using data from Taiwan's National Health Insurance database on pediatric patients hospitalized from 1997 to 2005 and having a CHD diagnosis before the age of three. Out of the 24,729 patients, 237 (0.96%) developed IE (cases). 4725 were randomly selected from the CHD patients who did not contract IE (*n* = 24,492; 99.04%) during the study period (1997–2010). The study found no correlation between dental work and IE contraction, with 13 of 237 patients (5.49%) having a history of dental procedures done in the last 6 months. Out of the 13 children who did have dental work within 6 months of IE contraction, 10 of them used AP before the dental procedure (76.92%), and 3 did not (23.08%). Thus, the adjusted OR for the incidence of IE in patients receiving AP before dental procedures was 1.31 (95% CI: 0.64, 2.66; *p* = 0.459) compared to the children without dental procedures whereas the adjusted OR for the incidence of IE without AP before dental procedures was 0.35 (95% CI: 0.11, 1.17; *p* = 0.088) compared to the adjusted OR in patients in whom no dental procedure was done. With these results, the study indirectly found AP unnecessary for CHD patients undergoing dental work.

**FIGURE 2:**
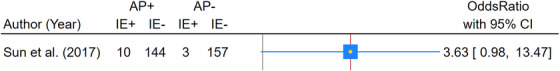
Visual graph of the only study (Sun et al., [15]) found in the present review (Outcome: IE. infective endocarditis). Abbreviations: AP−‐, antibiotic prophylaxis not given; AP+, antibiotic prophylaxis given; IE−‐, did not develop infective endocarditis; IE+, developed infective endocarditis.

**FIGURE 3:**
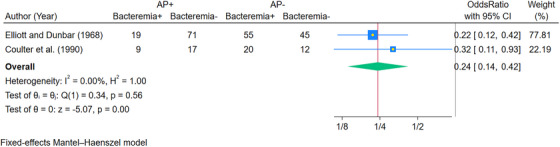
Forest plot for the occurrence of bacteremia with or without antibiotic prophylaxis in cardiac patients versus controls. Abbreviations: AP−‐, antibiotic prophylaxis not given; AP+, antibiotic prophylaxis given; Bacteremia−‐, blood culture negative after dental procedure; Bacteremia+, blood culture positive (defined as bacteremia) after dental procedure.

Due to one study evaluating IE as an outcome, a meta‐analysis could not be performed for IE, although we intended to perform the quantitative synthesis as per our review protocol. Additionally, we were unable to conduct a sensitivity analysis or a subgroup analysis according to different variables (for the dosage and type of antibiotics, type/severity of heart or systemic conditions [mild, moderate, or high risk], and duration of follow‐up). Publication bias using funnel plots also could not be done due to the lack of primary studies on the topic. There were 3.63 times higher odds of developing IE after the dental procedure when antibiotics were administered compared to when no antibiotics were administered, although it was not statistically significant (OR: 3.63, 95% CI: 0.98, 13.47; *p* = 0.05) [Figure 2]. A meta‐analysis of only two studies that evaluated bacteremia as an outcome was attempted using the fixed effects Mantel–Haenszel model. There were fewer odds of developing bacteremia after the dental procedures if prophylactic antibiotics were administered (OR: 0.24, 95% CI: 0.14, 0.42; *p* < 0.05) [Figure 3]. The heterogeneity was low, as evident by the *I* ^2^ value of 0.00% (*p* = 0.56).

### 3.5 Certainty of Assessment

The certainty of evidence was evaluated using the Grading of Recommendations Assessment, Development, and Evaluation (GRADE) approach [18] using GRADEpro GDT software, which calculates the evidence based on study design, risk of bias, inconsistency, indirectness, imprecision, and other factors (like the strength of association). Table 4 shows the GRADE evidence profile of the parameters studied. There was very low certainty of evidence that could determine whether AP did or did not lower the risk of contracting IE in pediatric patients with congenital cardiac disorders undergoing dental procedures, and very low evidence that the AP was effective in reducing bacteremia. The main reasons for downgrading the evidence were related to study designs and suboptimal information criteria due to a limited number of studies.

**TABLE 4:** GRADE [18] evidence profile (using GRADEpro GDT).

												Summary of findings		
	Certainty assessment							Number of exposed (AP+)		Number of controls (AP−)		Effect		
Outcome	Number of studies	Study design	Risk of bias	Inconsistency	Indirectness	Imprecision^a^	Other considerations^b^	IE+ (*N*)	IE− (*N*)	IE+ (*N*)	IE− (*N*)	Relative (95% CI)	Absolute (95% CI)	Certainty of evidence
IE	1 [15]	Case‐control	Not serious	Not serious	Not serious	Serious	None	10	144	3	157	OR 3.63 (0.98, 13.47)	0 fewer per 1000 (from 0 fewer to 0 fewer)	⨁◯◯◯ **Very low**
Bacteremia	2 [16, 17]	Case‐control	Serious	Not serious	Not serious	Serious	None	28	88	75	57	OR 0.24 (0.14, 0.42)	0 fewer per 1000 (from 0 fewer to 0 fewer)	⨁◯◯◯ **Very low**

Abbreviations: CI, confidence intervals; *N*, Number.

^a^ Imprecision: Downgraded as the OIS (optimal information size) criteria was not met: 300 events in case of dichotomous outcomes and 400 sample size in case of continuous outcomes. Moreover, the confidence intervals did not overlap [18].

^b^ OR of 3–6 was considered a strong association, whereas OR above six was considered a very strong association between the exposure and the event, and the level of evidence was upgraded accordingly. OR less than three was not considered strong enough and thus was not upgraded.

## 4 Discussion

The present systematic review was undertaken to generate evidence regarding the use of AP before dental procedures in children with congenital heart disease for the prevention of IE. There was very low evidence to conclude that AP before dental procedures is effective in reducing the risk of IE or bacteremia in children with congenital heart disease due to insufficient studies that could be included. Even though the empirical evidence for AP for preventing IE in pediatric patients with CHD is inconclusive, its continued recommendation for high‐risk individuals is probably based on the serious clinical consequences of IE, understanding of the pathophysiology and prevention of IE, and expert consensus due to practical and ethical limitations in conducting large studies. Despite that AP might not be indicated in all congenital cardiac diseases, we intentionally adopted wide inclusion criteria to identify any literature from the past when AP was deemed appropriate or to capture the studies from different settings with different prophylaxis guidelines. Although RCTs were also included in our PICOS criteria, none were identified. Conducting such studies would raise significant ethical concerns, particularly if the control group—comprising high‐risk patients with congenital heart disease—were withheld prophylactic antibiotics before dental procedures and then followed longitudinally for the development of IE. Lack of primary studies on this topic has also been highlighted by an earlier systematic review that evaluated the role of prophylactic antibiotics in the prevention of IE in adults. The review included a single study and found the evidence to be *“very low,”* which is in agreement with the current review [19]. Another review, with wider inclusion criteria, included case‐control studies, cohort studies, time‐trend studies (to evaluate the change in prevalence/incidence of IE), and also studies to evaluate AP on bacteremia, also concur with our results that the evidence for the use of AP before dental procedures to prevent IE is limited due to poor study designs [20].

Nevertheless, the importance of AP for preventing IE in high‐risk cardiac patients has been stressed in most of the guidelines, and the present systematic review sheds light on the evidence of AP before dental procedures in pediatric patients with congenital cardiac disorders. A systematic review appraised the quality of AP guidelines (by the European Society of Cardiology, AHA, National Institute of Health and Care Excellence, and Japanese Circulation Society) after dental procedures and concluded that three out of four guidelines supported the use of prophylaxis for preventing IE, as the benefits of prophylaxis outweigh the risk of IE and antibiotic resistance [21]. The review conducted on adults found that the odds of IE in high cardiac risk adults were 0.39 when AP was given compared to when no AP was given, suggesting a protective role for antibiotics, although not statistically significant (*p* = 0.11) [19]. However, in the present review, the odds of IE with AP were 3.63 compared to those without AP in high‐risk cardiac pediatric patients (*p* = 0.05). Contrastingly, a recent meta‐analysis found that AP was associated with a significantly lower risk of IE after dental procedures (pooled risk ratio: 0.41; 95% CI: 0.29, 0.57) only in high‐risk cardiac patients but not in low or moderate cardiac risk [22]. The disagreement in this review might be due to the inclusion of different study designs (case‐control, cohorts, time trends) and the review's focus on the adult population with other cardiac disorders, like heart valves and comorbidities, due to the aging population. This review did not pool together time trend studies with case‐control and cohort studies and included all published pediatric studies. A disagreement was found within the time‐trends studies, which are epidemiologic studies affected by multiple confounders and for which heterogeneous statistical analyses have been used. AP has also been shown to be cost‐effective in patients with a risk of IE, especially high‐risk patients; however, this result is based on a decision‐analytical cost‐effectiveness model [23]. Nevertheless, changing or modifying guidelines for AP would require immense research, which might not be ethically feasible due to the seriousness of IE in children who might not receive prophylaxis. The current review is an attempt to add to the literature and would probably encourage scientific curiosity and further research.

Based on the two studies included in our review that studied bacteremia as an outcome in pediatric patients with CHD, we can conclude that AP can prevent bacteremia after dental procedures in patients with cardiac disorders compared to no AP in healthy patients, which is in agreement with previous systematic reviews [24, 25]. However, both the included studies are old, and thus the antibiotic regimens are outdated, which raises concerns about the external validity of these results in the present day. It is noteworthy to mention that bacteremia occurs after dental procedures and can peak after 1.5–5 min of the dental procedures or routine oral hygiene procedures like toothbrushing and flossing [1]; however, there are no studies that have validated bacteremia as a reliable surrogate marker for IE [20]. Thus, future studies with appropriate study designs could also study whether bacteremia is responsible for IE. Also, it is not known if the increased risk of bacteremia is driven by the underlying cardiac disease rather than AP, which might act as a strong confounder. Ideally, a RCT with an adequate sample size might be able to evaluate the effectiveness of AP before dental procedures in high‐risk cardiac patients to prevent IE, but practically, such an RCT will require a control group with high cardiac risk that does not receive antibiotics and thus might not be ethically feasible.

One of the limitations of the present review is the lack of primary studies on the topic, which has been a limitation for other similar reviews. Although the only included study [15] evaluating IE as an outcome was a nested case‐control design, it had a large sample size. Thus, the review adds to the current understanding of the topic and warrants a scientific discussion and further research on the efficacy of AP in the prevention of IE in high‐risk cardiac patients. A robust methodology, strict inclusion criteria, and the use of a GRADE evidence profile are some of the strengths of the present review. Despite the limited research on the topic, most of the current guidelines recommend or endorse the use of AP before dental procedures to prevent IE in high‐risk cardiac patients, which underscores the dire need for future research on the topic. This is probably due to the severity of IE and its associated mortality, and weighing the benefits of AP against the harms of AP, even if the risk of the disease is low. Future research with appropriate study designs and sample sizes should evaluate the effectiveness of AP on the prevention of IE in children after dental procedures and the cost‐effectiveness and risk of antibiotic resistance in preventing IE.

## 5 Conclusions

Within the limitations of the present systematic review, we can conclude that there was very low evidence that AP before dental procedures was effective in preventing IE in pediatric patients with congenital cardiac disorders. There is very low certainty of evidence that antibiotics might have a role in preventing bacteremia after dental procedures in pediatric patients with congenital cardiac disorders. There were no studies that evaluated the role of AP in preventing IE in high cardiac risk patients. Thus, due to the lack of empirical evidence, it is difficult to make a recommendation for or against the use of AP in the prevention of IE in pediatric patients with congenital cardiac disorders (low, moderate, or high risk). Future research should evaluate the clinical validity of bacteremia as a risk marker for IE and large‐scale studies to assess the role of prophylaxis in the prevention of IE in children.

## Author Contributions

Conceptualization, methodology, validation, investigation, and writing (original draft): C. Loper, J. Baskette, and Divesh Sardana. Supervision and project administration: T. R. Fagan and Divesh Sardana. Writing (review and editing): T. R. Fagan. Formal analysis, data curation, and funding acquisition: Divesh Sardana. All the authors gave final approval of the article before submission and agreed to be accountable for all aspects of the work, ensuring integrity and accuracy.

## Funding

The study was funded by the J. Dean Robertson Society and the Delta Dental of Oklahoma Foundation through the Student Research Program of the University of Oklahoma College of Dentistry.

## Conflicts of Interest

The authors declare no conflicts of interest.

## Supporting information




**Supporting File 1**: scd70112‐sup‐0001‐SuppMat.xlsx

**Supporting File 2**: scd70112‐sup‐0002‐SuppMat.xlsx

## 1 Ovid MEDLINE(R) ALL 1946 to May 09, 2024

**Table:**

S No.	Keywords	Number of yields
1.	exp Dentistry/	440512
2.	(dental procedur* or oral procedur* or maxillofacial procedur* or maxillofacial surgery or operative dentistry or dental care or oral surgery or dental surgery).tw.	35395
3.	1 or 2	455486
4.	exp Antibiotic Prophylaxis/	15792
5.	exp Pre‐Exposure Prophylaxis/	5285
6.	(antibiotic prophylaxis or prophylaxis or prevention or antibiotic premedic* or antimicrobial or antibiotic*).tw.	1343098
7.	4 or 5 or 6	1347673
8.	3 and 7	18693
9.	exp clinical trials as topic/ or intervention studies/	391721
10.	Random Allocation/	107192
11.	random*.tw.	1514066
12.	placebo.tw.	254798
13.	exp Case‐Control Studies/ or case control.mp.	1556266
14.	exp Cohort Studies/ or cohort.mp.	2961703
15.	9 or 10 or 11 or 12 or 13 or 14	4812142
16.	8 and 15	4625
17.	exp Endocarditis/	31333
18.	(endocarditis or endocarditides).tw.	40224
19.	Endocarditis, Subacute Bacterial.mp. or exp Endocarditis, Bacterial/ or exp Endocarditis, Subacute Bacterial/ or exp Endocarditis/	31336
20.	17 or 18 or 19	46092
21.	16 and 20	172
22.	exp Infant/ or pediatrics.mp. or exp Pediatrics/ or exp Child, Preschool/	1816423
23.	exp Child/ or paediatric.mp. or exp Child, Preschool/	2228621
24.	children.mp. or exp Child/	2553883
25.	22 or 23 or 24	3171763
26.	21 and 25	**30**

## 1 Embase Classic+Embase 1947 to 2024 May 09

**Table:**

S No.	Keywords	Number of yields
1.	exp Dentistry/	143502
2.	(dental procedur* or oral procedur* or maxillofacial procedur* or maxillofacial surgery or operative dentistry or dental care or oral surgery or dental surgery).tw.	41007
3.	1 or 2	178988
4.	exp Antibiotic Prophylaxis/	41369
5.	exp Pre‐Exposure Prophylaxis/	10456
6.	(antibiotic prophylaxis or prophylaxis or prevention or antibiotic premedic* or antimicrobial or antibiotic*).tw.	1852842
7.	4 or 5 or 6	1871136
8.	3 and 7	10842
9.	exp clinical trials as topic/ or intervention studies/	517737
10.	Random Allocation/	94329
11.	random*.tw.	2077257
12.	placebo.tw.	381545
13.	exp Case‐Control Studies/ or case control.mp.	304810
14.	exp Cohort Studies/ or cohort.mp.	1738434
15.	9 or 10 or 11 or 12 or 13 or 14	4339463
16.	8 and 15	1623
17.	exp Endocarditis/	66054
18.	(endocarditis or endocarditides).tw.	57519
19.	Endocarditis, Subacute Bacterial.mp. or exp Endocarditis, Bacterial/ or exp Endocarditis, Subacute Bacterial/ or exp Endocarditis/	66111
20.	17 or 18 or 19	74995
21.	16 and 20	136
22.	exp Infant/ or pediatrics.mp. or exp Pediatrics/ or exp Child, Preschool/	1952948
23.	exp Child/ or paediatric.mp. or exp Child, Preschool/	3649132
24.	children.mp. or exp Child/	3952577
25.	22 or 23 or 24	4038564
26.	21 and 25	**12**

**PubMed (726 results)**

((((antibiotic prophylaxis) OR (Pre‐Exposure Prophylaxis/) OR (antimicrobial)) AND ((clinical trials) OR (Randomized) OR (random) OR (case control) OR (cohort) OR (longitudinal))) AND ((infective endocarditis) OR (endocarditides) OR (septicemia OR bacteremia) OR (Endocarditis, Subacute Bacterial))) AND ((dentistry) OR (dental) OR (Oral health) OR (mouth))

**Scopus (306 results)**

((((antibiotic prophylaxis) OR (Pre‐Exposure Prophylaxis/) OR (antimicrobial)) AND ((clinical trials) OR (Randomized) OR (random) OR (case control) OR (cohort) OR (longitudinal))) AND ((infective endocarditis) OR (endocarditides) OR (septicemia OR bacteremia) OR (Endocarditis, Subacute Bacterial))) AND ((dentistry) OR (dental) OR (Oral health) OR (mouth))

## 1 CINAHL

**Table:**

S. No.	Keywords	Number of yields
	S3 AND S4	**32**
S4	(MH “Mouth”) OR “dental OR mouth OR oral”	4084
S3	S1 OR S2	13563
S2	(MH “Endocarditis+”) OR (MH “Endocarditis, Bacterial”) OR “(infective endocarditis) OR (subacute bacteria endocarditis)”	5123
S1	(MH “Bacteremia”) OR “bacteremia”	8814

## 1 Web of Science

**Table:**

S. No.	Keywords	Number of yields
1.	ALL = ((infective endocarditis) OR (bacteremia) OR (subacute bacterial endocarditis) OR (Endocarditis prophylaxis))	66223
2.	ALL = ((pediatrics) OR (child) OR (children) OR (pediatrics) OR (infant) OR (neonate) OR (adolescent))	3408988
3.	#1 AND #2	10120
4.	ALL = ((dentistry) OR (dental) OR (mouth) OR (oral))	1828287
5.	#3 AND#4	**875**
